# Optimized Degradation and Inhibition of α-glucosidase Activity by *Gracilaria lemaneiformis* Polysaccharide and Its Production In Vitro

**DOI:** 10.3390/md20010013

**Published:** 2021-12-22

**Authors:** Xiaoshan Long, Xiao Hu, Shaobo Zhou, Huan Xiang, Shengjun Chen, Laihao Li, Shucheng Liu, Xianqing Yang

**Affiliations:** 1 Key Laboratory of Aquatic Product Processing, Ministry of Agriculture and Rural, South China Sea Fisheries Research Institute, Chinese Academy of Fishery Sciences, Guangzhou 510300, China; longxiaoshan1992@163.com (X.L.); xianghuan@scsfri.ac.cn (H.X.); chenshengjun@scsfri.ac.cn (S.C.); lilaihao@scsfri.ac.cn (L.L.); 2 Co-Innovation Center of Jiangsu Marine Bio-Industry Technology, Jiangsu Ocean University, Lianyungang 222005, China; 3 College of Food Science and Technology, Guangdong Ocean University, Guangdong Provincial Key Laboratory of Aquatic Products Processing and Safety, Guangdong Provincial Engineering Technology Research Center of Marine Food, Guangdong Province Engineering Laboratory for Marine Biological Products, Zhanjiang 524088, China; Lsc771017@163.com; 4 School of Life Sciences, Institute of Biomedical and Environmental Science and Technology, University of Bedfordshire, Luton LU1 3JU, UK; shaobo.zhou@beds.ac.uk; 5 Collaborative Innovation Center of Provincial and Ministerial Co-construction for Marine Food Deep Processing, Dalian 116034, China

**Keywords:** *Gracilaria lemaneiformis*, polysaccharide, degradation optimization, chemical characteristics, hypolipidemic activity, *α*-glucosidase

## Abstract

*Gracilaria lemaneiformis* polysaccharide (GLP) exhibits good physiological activities, and it is more beneficial as it is degraded. After its degradation by hydrogen peroxide combined with vitamin C (H_2_O_2_-Vc) and optimized by Box–Behnken Design (BBD), a new product of GLP-HV will be generated. While using GLP as control, two products of GLP-H (H_2_O_2_-treated) and GLP-V (Vc-treated) were also produced. These products chemical characteristics (total sugar content, molecular weight, monosaccharide composition, UV spectrum, morphological structure, and hypolipidemic activity in vitro) were assessed. The results showed that the optimal conditions for H_2_O_2_-Vc degradation were as follows: H_2_O_2_-Vc concentration was 18.7 mM, reaction time was 0.5 h, and reaction temperature was 56 °C. The total sugar content of GLP and its degradation products (GLP-HV, GLP-H and GLP-V) were more than 97%, and their monosaccharides are mainly glucose and galactose. The SEM analysis demonstrated that H_2_O_2_-Vc made the structure loose and broken. Moreover, GLP, GLP-HV, GLP-H, and GLP-V had significantly inhibition effect on α-glucosidase, and their IC_50_ value were 3.957, 0.265, 1.651, and 1.923 mg/mL, respectively. GLP-HV had the best inhibition effect on α-glucosidase in a dose-dependent manner, which was the mixed type of competitive and non-competitive. It had a certain quenching effect on fluorescence of α-glucosidase, which may be dynamic quenching.

## 1. Introduction

*Gracilaria lemaneiformis* belongs to *Rhodophyta* Phylum, *Gigartinales* Order, *Gracilariaceae* Family, and *Gracilaria* Genus, which is a kind of economic red algae, used as agar, feed, food, and drug resources [1,2]. The mariculture yield of *gracilaria* accounts for 10% of the algae production, second only to kelp. The main producing areas of *Gracilaria lemaneiformis* are the province of Fujian (75.5%), Guangdong (12.3%), and Shandong (12.3%) in China, and it is a traditional seaweed used both as medicine and food [3]. Polysaccharide is the main component of *Gracilaria lemaneiformis*, which has the physiological activities of hypoglycemic, hypolipidemic, hypotensive, anti-obesity, and antineoplastic effects [4,5,6,7,8,9]. However, the molecule and viscosity of *Gracilaria lemaneiformis* polysaccharide (GLP) are large, and the structure is complex, which is not conducive to the digestion and absorption in human body, and limits the application of polysaccharide. It has been said that polysaccharide with lower molecular weight presented better physiological effects after degradation [10,11,12]. Xu et al. [13] gained the degradation products of GLP with better tyrosinase inhibition and antioxidant abilities. Jin et al. [14] proved that oligosaccharides from *Gracilaria lemaneiformis* exhibited the protective effect on alcohol-induced hepatotoxicity.

It has been reported that degradation methods include physical [15,16,17] (ultrasonic, microwave, high temperature, high pressure, radiation), biological [12,18] (fermentation and enzyme), and chemical [11] (acid hydrolysis, reductant-oxidant) methods. The principle of physical degradation is to break the glyosidic bonds of polysaccharide by mechanical means, which has the advantages of being environmentally friendly, simple operation, controllable conditions, and low energy consumption. However, the degradation degree and the efficiency are low. It is usually used in combination with chemical methods [16]. Enzymes can selectively cut the specific glyosidic bond, which has the advantages of high efficiency and maintaining the structure of polysaccharide [19]. However, it has the disadvantages of specificity and high cost, which is unsuitable for a large number of degradations of polysaccharide [13]. Chemical degradation of polysaccharide mainly uses the chemical reagents to destroy glyosidic bonds to achieve the purpose of degradation, which is more rapid and low-cost [17]. In the acid degradation method, some inorganic acids, such as phosphoric acid and hydrochloric acid, are adopted to cause the glyosidic bond breakage, which degrade the polysaccharide into low molecular substances, and cause environmental pollution, resulting in by-products and low purity of degradation products [13]. Oxidants (such as H_2_O_2_) use free radicals to attack glyosidic bonds of polysaccharide to degrade, with non-toxic, convenient, inexpensive and without by-products, which is suitable to apply in the industry. Nevertheless, the sensitivity of glyosidic bonds at various positions of polysaccharide to H_2_O_2_ is different, therefore it is necessary to explore the conditions of H_2_O_2_ degradation, including H_2_O_2_ concentration, reaction time, temperature, etc., [20]. Alone, H_2_O_2_ treatment has a low oxidative degradation efficiency, while H_2_O_2_ can produce higher hydroxyl radicals with the assistance of other reagents (Vc, Cu^2+^ and Fe^2+^) or means (ultrasound, microwave, radiation) [17]. Vc, Cu^2+^ and Fe^2+^ have strong reducibility that can react with H_2_O_2_ to produce HO^2^- and OH-. These free radicals degrade polysaccharide by attacking glyosidic bonds [16,21]. Chen et al. [10] adopted H_2_O_2_-Vc to degrade *Grateloupia livida* polysaccharide, and found that H_2_O_2_-Vc degradation is fast, effective, and beneficial to enhance the antioxidant activity of polysaccharide. It also has been found that the degradation of GLP treated by radiation-H_2_O_2_ promoted the better activity [11]. Compared with other methods, the effect of free radicals on the glyosidic bond is stronger, the degree of degradation is better, causes more reducing sugar to produce, and reduces the molecular weight of polysaccharide [11,15,19]. 

Studies have shown that α-glucosidase is one of the enzymes that hydrolyze polysaccharide. α-glucosidase inhibitors can effectively delay the hydrolysis and absorption of carbohydrate by inhibiting α-glucosidase at the brush edge of the small intestinal mucosa, thus improving the symptoms of hyperglycemia [22,23]. Wen et al. [24] proved that GLP found the obvious hypoglycemic activity. Research has explored that GLP and its degradation expressed the hypoglycemic effect by inhibiting α-glucosidase activity, and the polysaccharide degradation had better inhibition effect [25]. According to previous reports, the inhibitory activity of polysaccharide on α-glucosidase was related to a number of factors, such as molecular weight, monosaccharide composition, and other structures [26]. 

In the present study, the degradation conditions of H_2_O_2_-Vc, the structure of degradation products of GLP, and its inhibition effect of α-glucosidase were explored. 

## 2. Results

### 2.1. Optimized Degradation of Gracilaria Lemaneiformis Polysaccharide

#### 2.1.1. Results of Single Factor Experiment

**Figure 1:**
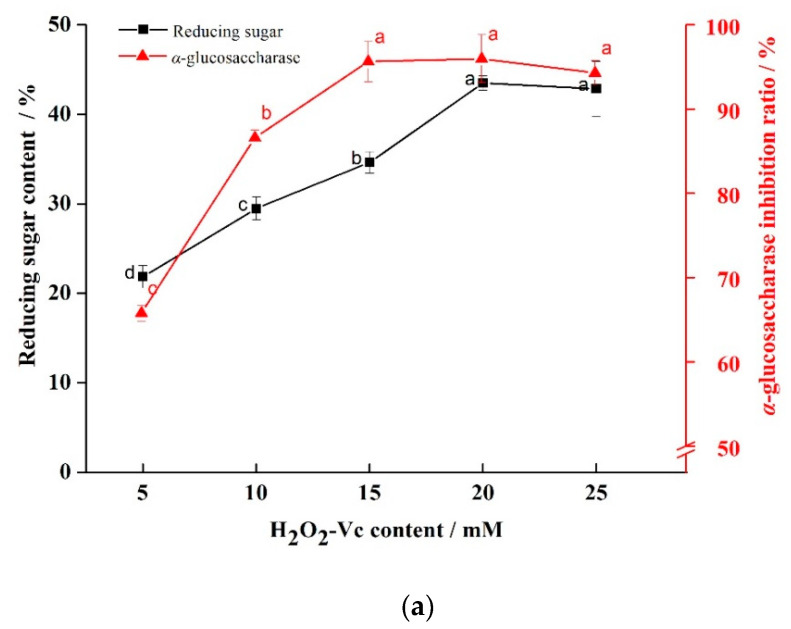

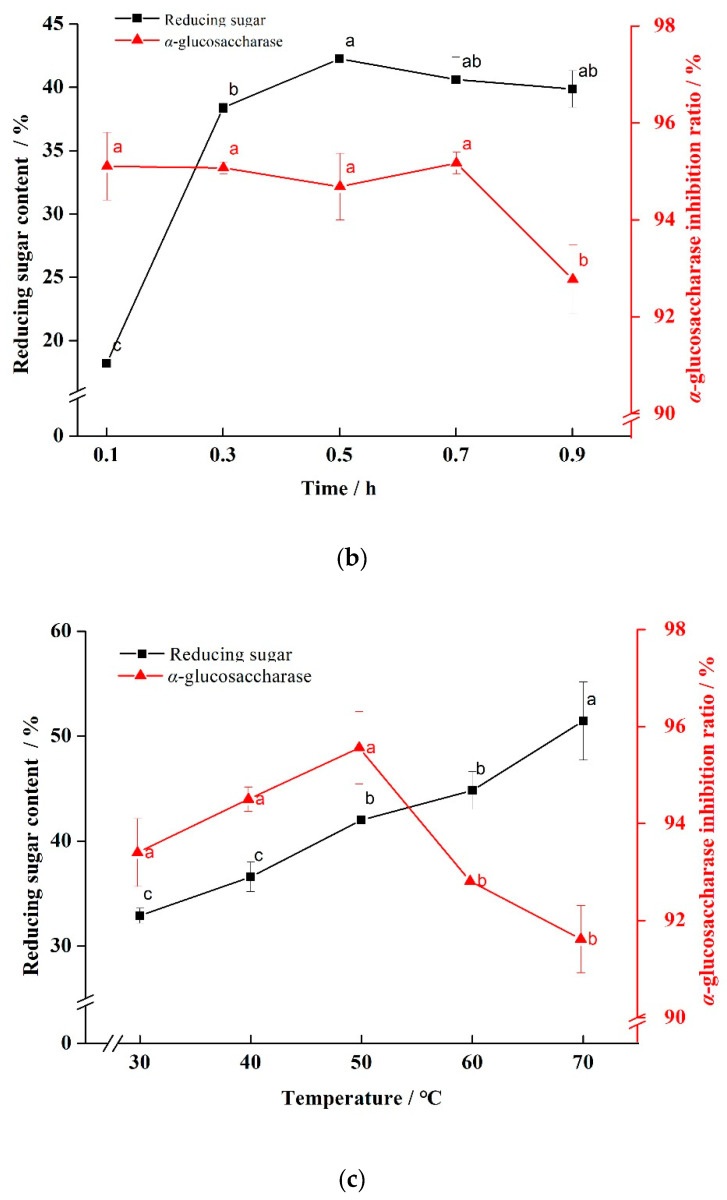
Effect of H_2_O_2_-Vc concentration, time, and temperature on degradation. Note: (**a**) Effect of H_2_O_2_-Vc concentration on degradation; (**b**) effect of time on degradation; (**c**) effect of temperature on degradation; a, b, and c represent significant differences among groups, and *p* < 0.05 indicates significant differences.

To research the effect of H_2_O_2_-Vc concentration on degradation of GLP, the experiment was carried out under the conditions of temperature of 50 °C, reaction time of 0.5 h and H_2_O_2_-Vc concentration of 5, 10, 15, 20, and 25 mM, respectively. Reducing sugar content and α-glucosidase inhibition rate were taken as the screening indexes. From Figure 1a, both reducing sugar content and α-glucosidase inhibition rate were the highest when the concentration of H_2_O_2_-Vc concentration was 20 mM.

In order to study the effect of time on degradation, the concentration of H_2_O_2_-Vc was 20 mM (attained from Figure 1a), the temperature was 50 °C, and the time was 0.1, 0.3, 0.5, 0.7, and 0.9 h. The reducing sugar content and α-glucosidase inhibition rate were taken as indexes. The results (Figure 1b) showed that the reducing sugar content and α-glucosidase inhibition rate were the highest when the time was 0.5 h. 

On the basis of the above experiments (H_2_O_2_-Vc concentration was 20 mM and time was 0.5 h), temperature of 30, 40, 50, 60, and 70 °C were selected to explore the influence of temperature on the degradation. The results showed that the reducing sugar content and α-glucosidase inhibition rate were higher when the temperature was 50 °C (as seen in Figure 1c).

The above results indicated that H_2_O_2_-Vc concentration, time, and temperature all had certain effects on degradation of GLP. The reducing sugar content and α-glucosidase inhibition rate were the best when H_2_O_2_-Vc was 20 mM, time was 0.5 h and temperature was 50 °C, respectively. 

#### 2.1.2. Results of Response Surface Experiment

##### Establishment of Regression Model

According to the single factor experiment results, H_2_O_2_-Vc concentration (A), time (B) and temperature (C), were selected for response surface analysis. The codes “−1”, “0”, and “1” severally represented the three levels of each factor (Table 1). A total of 17 experiment groups were designed, and the reducing sugar was the response value of the degradation products of GLP (Table 2). The Box–Behnken Design (BBD) principle of Design-Expert 8.0.6 was used to design and implement the corresponding surface method, and the response surface results were fitted. Table 2 presented the response surface experimental design scheme and results, and the regression equation was Y = 40.81 − 1.35A + 0.81B + 1.98C + 0.85AB − 0.84AC − 0.5BC − 3.28A^2^ − 1.87B^2^ − 1.81C^2^.

##### Regression Model Analysis

In Table 3, *F* value of this model was 63.28, *p* value < 0.0001 (extremely significant), and the misfitting item was 0.8223 > 0.05 (insignificant). The indexes (R^2^ = 0.9879 and R^2^adj = 0.9722) indicated that the regression equation could accurately reflect the influence of various factors on the degradation of GLP, which can be used for the analysis and prediction of the degradation of GLP by H_2_O_2_-Vc. The values of A, B, C, A^2^, B^2^, and C^2^ were all less than 0.0001, showing extremely significant difference. According to the value of *F*, the influence order of these three factors was C > A > B. Temperature had the greatest influence on the degradation of GLP, while time had the least influence. The *p* value of AB and AC were less than 0.01, while the *p* value of BC was greater than 0.05, indicating that the interaction between H_2_O_2_-Vc concentration and time was obvious, and the interaction between H_2_O_2_-Vc concentration and temperature was also significant, while the interaction between time and temperature was not significant.

##### Analysis of Model Interaction Items

**Figure 2:**
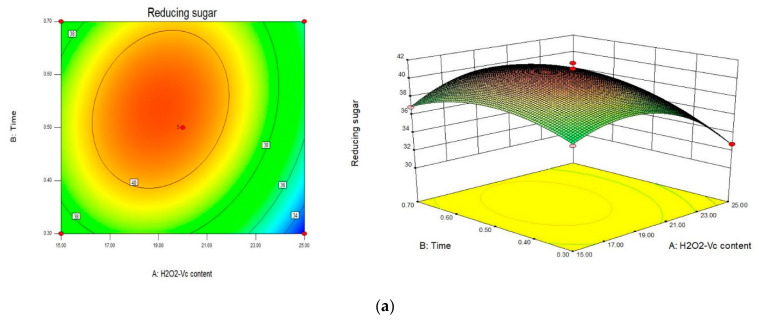

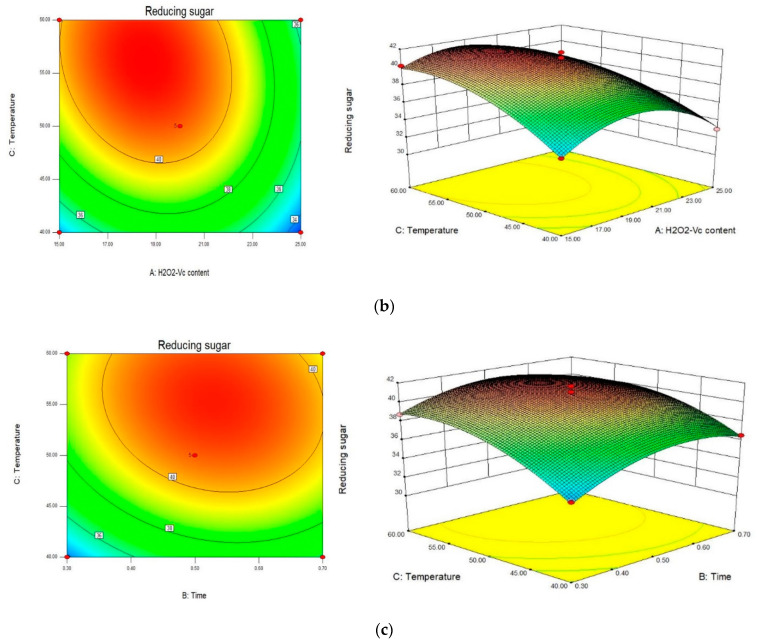
Response surface diagram and contour diagram of interaction of various factors on polysaccharide degradation. (**a**) The interaction between concentration and time; (**b**) The interaction between concentration and temperature; (**c**) The interaction of time and temperature.

Figure 2 showed the contour map and response surface map of the degradation products of GLP. The contour maps of Figure 2a,b were oval, and (c) was circular. The response surface slope of (a) and (b) were larger than (c). These results indicated that H_2_O_2_-Vc concentration had significant interaction with reaction time, and H_2_O_2_-Vc concentration had remarkable interaction with temperature, while the interaction between reaction time and temperature was not obvious, which was consistent with the results of regression model analysis.

##### Response Surface Optimization and Validation

According to Design-Expert 8.0.6, the optimal process conditions of degradation were as follows: H_2_O_2_-Vc concentration was 18.64 mM, reaction time was 0.51 h, and reaction temperature was 56.03 °C. On the basis of the actual situation, the process parameters were adjusted. After adjustment, the concentration of H_2_O_2_-Vc was 18.7 mM, the reaction time was 0.5 h, and the reaction temperature was 56 °C. Under these conditions, the reducing sugar content of the degradation product was 45.16%. The measured value reached 41.62% (the predicted value), which proved that the process conditions of response surface optimization were accurate and reliable, and the model was suitable. The deviation between the actual measured value and the predicted value was less than 5%, indicating that the response surface optimization scheme was reliable. 

### 2.2. The Content of Total Sugar, Reducing Sugar and Protein

In Table 4, the total sugar content of GLP, GLP-HV, GLP-H, GLP-V were 98.77%, 98.43%, 97.28%, and 97.5%, respectively, which showed no significant difference among them (*p* > 0.05). It showed us that extraction method of hot water and alcohol precipitation were more suitable for the extraction of GLP. In addition, the total sugar content of polysaccharide degradation products did not decrease. The total sugar content was only 66.68% treated by critic acid [24], which was far lower than the polysaccharide obtained by the method of water extraction and alcohol precipitation in this experiment. The total sugar content severally was 91.4% with the method of amylase-assisted extraction from Wu et al. [27]. Li et al. [4] got 59% total sugar content, illustrating that the polysaccharide obtained by acid extraction has more impurities. Moreover, the carbohydrate content was 72.06% extracted with cold water [6]. In the present experiment, the total sugar content of GLP obtained by hot water extraction was relatively high, compared with other methods, and the content did not decrease after degradation.

The reducing sugar of GLP was 2.47%, which enhanced the obvious degradation by H_2_O_2_-Vc (GLP-HV was 46.92%) or Vc (GLP-V was 50.2%). However, GLP-H was only 1.95%, significantly lower than GLP. The results proved that both H_2_O_2_-Vc-treated and Vc-treated promoted the production of reducing sugar, and H_2_O_2_-treated declined the reducing sugar content. The reducing sugar of GLP attained by Gong et al. [11] was 1.2%, lower than that of this experiment, and the content was diminished after treating with H_2_O_2_, which was consistent with the experimental results of this study. H_2_O_2_-Vc and Vc broke the glycosidic bonds, exposing more reducing sugars of polysaccharide. H_2_O_2_ may react with reducing groups on the sugar chain, declining the content of reducing sugar. When Vc was added, a redox reaction occurred between Vc and H_2_O_2_ to produce free radical, which promoted the breaking of glyosidic bonds and induced more reducing sugar. There was still a small amount of H_2_O_2_ that reacted with the reducing group, so the reducing sugar of GLP-HV was a little less than GLP-V.

The protein content of untreated *Gracilaria lemaneiformis* was 15.7 ± 0.0018%, detected by the Kjeldahl method. After treated by ethanol and papain, the protein of GLP was undetected with Coomassie Bright Blue method kit, which demonstrated that the protein was removed during the extraction of GLP. Liu et al. [28] detected the protein content of GLP treated by hot water was 0.98%. The protein of GLP attained by hot citric acid extraction was 1.47% [29]. Moreover, the protein content of GLP was 0.7% (enzyme extraction) [27], 1.6% (acid extraction) [4], 0.28% (extracted with cold water) [6]. From the above analysis, it can be seen that GLP obtained by other methods contain a small amount of protein, which can be removed with the method in this experiment. 

### 2.3. Monosaccharide Composition

**Figure 3:**
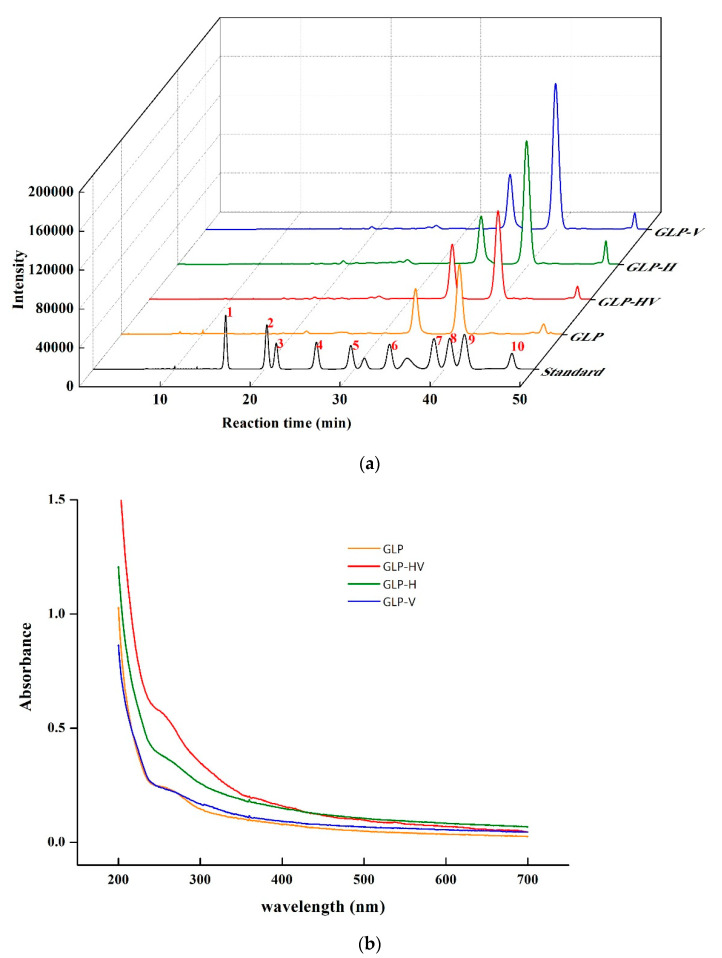

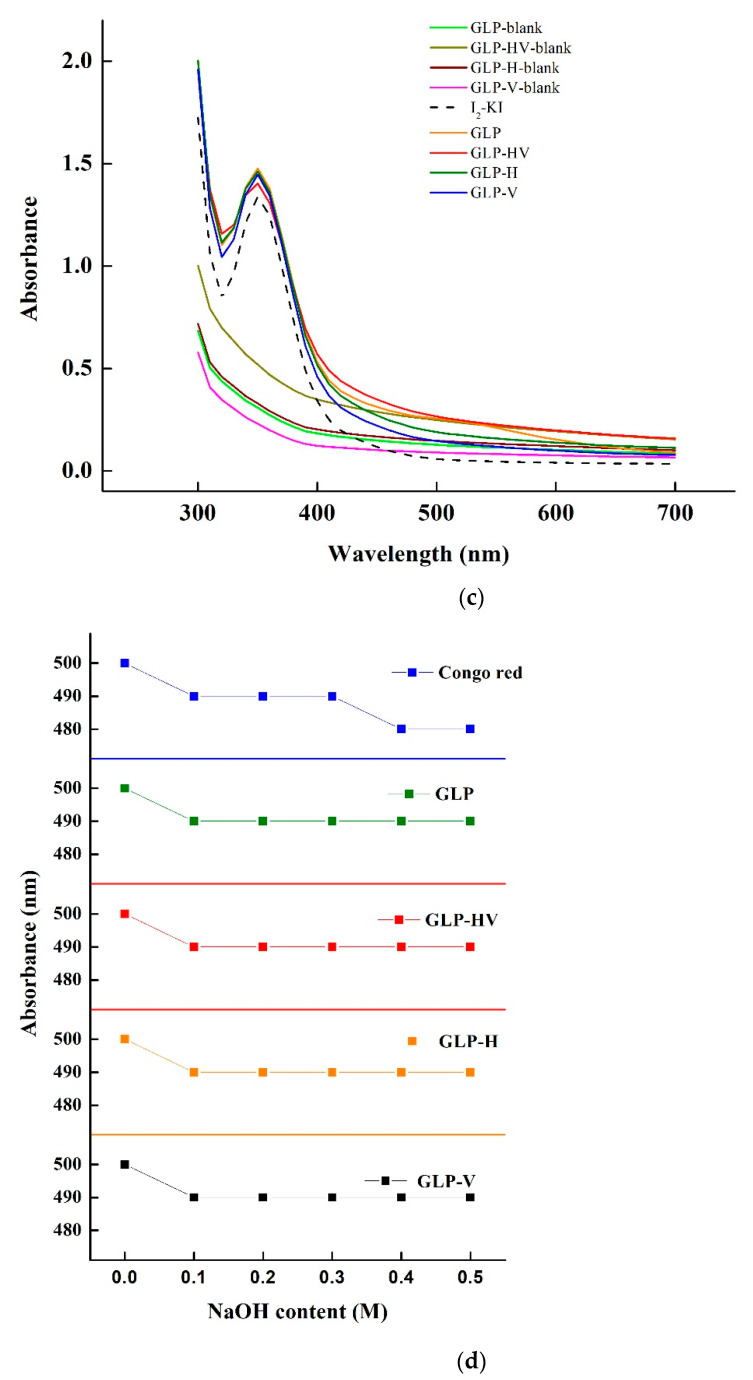
The monosaccharide composition, UV-visible spectroscopy, I_2_-KI test, Congo red test of GLP and its degradation products. Note: (**a**) The monosaccharide composition of GLP, GLP-HV, GLP-H, GLP-V; the number of 1–10 mean as follow: 1-Mannose, 2-Ribose,3-Rhamnose, 4-Glucuronic acid, 5- Galacturonic acid, 6-Glucose, 7-Galactose, 8-Xylose,9-Arabinose,10-Fucose (**b**) The UV-visible spectroscopy analysis of GLP, GLP-HV, GLP-H, GLP-V; (**c**) The I_2_-KI test of GLP, GLP-HV, GLP-H, GLP-V; (**d**) The Congo red test of GLP, GLP-HV, GLP-H, GLP-V.

As exhibited in Figure 3a, the main monosaccharide components of polysaccharide and its degradation products were glucose and galactose, accompanied by a small amount of mannose, ribose, glucuronic acid, galacturonic acid, arabinose, xylose, and fucose. GLP contained 34.35% glucose and 57.37% galactose, respectively, and GLP-HV included 33.37% glucose and 59.12% galactose. H_2_O_2_-Vc treatment slightly increased the content of galactose, while the glucose content declined a small amount, probably because there was a little bit more free radicals to attack the glyosidic bonds linking galactose. This result was consistent with Gong et al. [11]. The monosaccharide composition of GLP extracted by this method was relatively simple, mainly consisting of glucose and galactose, compared with previous studies. The content of glucose and galactose respectively were 4.76% and 21.1% from GLP extracted by Li et al. [4]. Moreover, the level of ribose and xylose were enhanced, but rhamnose content declined after degradation.

### 2.4. Molecular Weight 

The weight average molecular weight (Mw) of GLP was 14,78,524 Da (1478 kDa), which was higher than that of citric acid extraction (21.2 kDa and 31.5 kDa) [4,24], and it declined after degradation. In Table 5, the Mw of GLP-H and GLP-V were 1,329,838 and 1,000,630 Da, respectively, which was slightly lower than that of GLP. However, the Mw of GLP-HV was only 16,245 Da, which was much smaller than the molecular weight of GLP. It illustrated that the degradation level treated by H_2_O_2_-Vc was higher than alone H_2_O_2_ or Vc. Moreover, the value of Mw/Mn represents the molecular weight distribution, which can reflect the molecular weight distribution width and degree of polydispersity of polysaccharide. From Table 5, the Mw/Mn of GLP, GLP-HV, GLP-H, GLP-V were 144.24, 2.43, 75.96, and 47.15, respectively, which explained the GLP-HV performing the smallest distribution width, uniform distribution, and small dispersity. It was reported that the Mw of GLP was changed from 2.15 × 10^5^ to 1.22 × 10^5^ Da after degradation by fermentation [12], which demonstrated the degradation degree of H_2_O_2_-Vc was stronger than fermentation.

### 2.5. UV-Visible Spectroscopy

Four polysaccharide samples were respectively scanned under UV-visible spectroscopy at 200–700 nm. As shown in Figure 3b, GLP, GLP-HV, GLP-H, GLP-V showed no absorption at 260 nm and 280 nm, indicating that there were no nucleic acid and proteins before and after the degradation of the polysaccharide, which was consistent with the determination results of protein content in Table 4. 

### 2.6. I_2_-KI Test

The blank solution of GLP, GLP-HV, GLP-H, GLP-V had no absorption peak at 300–700 nm of UV-visible spectroscopy. These four polysaccharide solutions were mixed with I_2_-KI solution. After 10 min, I_2_-KI had the maximum absorption peak at 350 nm, and four polysaccharides also had the maximum absorption wavelength at 350 nm when mixed with I_2_-KI solution, while there was no absorption wavelength at 560 nm, indicating that the GLP, GLP-HV, GLP-H, and GLP-V contained no starch in the solution (Figure 3c). The GLP, degraded by UV-H_2_O_2_, also did not contain starch [11], which was consistent with the present research.

### 2.7. Congo Red Test

Congo red is an acidic dye soluble in water and alcohol. When it combines with the triple helix structure of polysaccharide to form a conjugate, its maximum absorption wavelength will be redshifted. However, when the concentration of NaOH increases, the complex formed by Congo red and the triple helix structure of polysaccharide will be destroyed, forming irregular curls, and the maximum absorption wavelength will first increase and then decrease [30]. From Figure 3d, GLP, GLP-HV, GLP-H, and GLP-V did not appear the phenomenon of redshifted, which were consistent with the change of pure Congo red. The results demonstrated that there was no triple helix structure in polysaccharide before and after the degradation.

### 2.8. Scanning Electron Microscope Analysis

**Figure 4:**
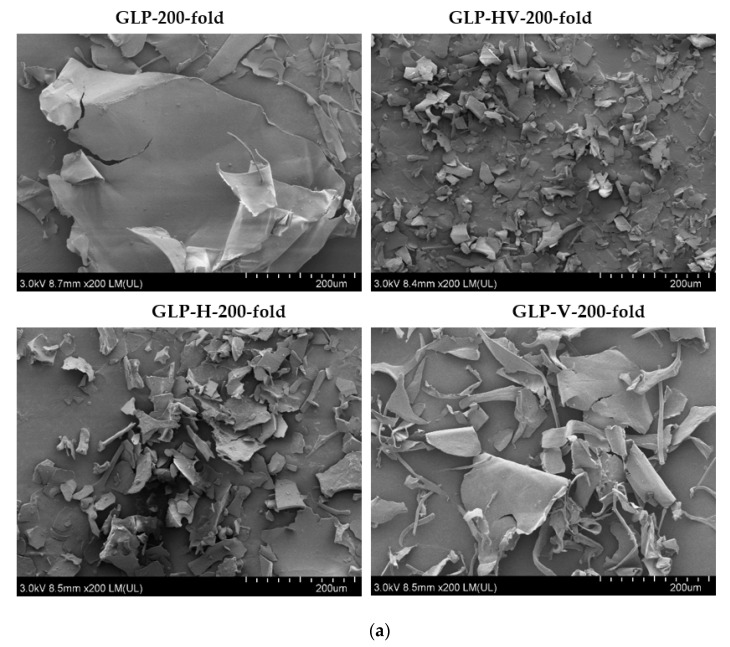

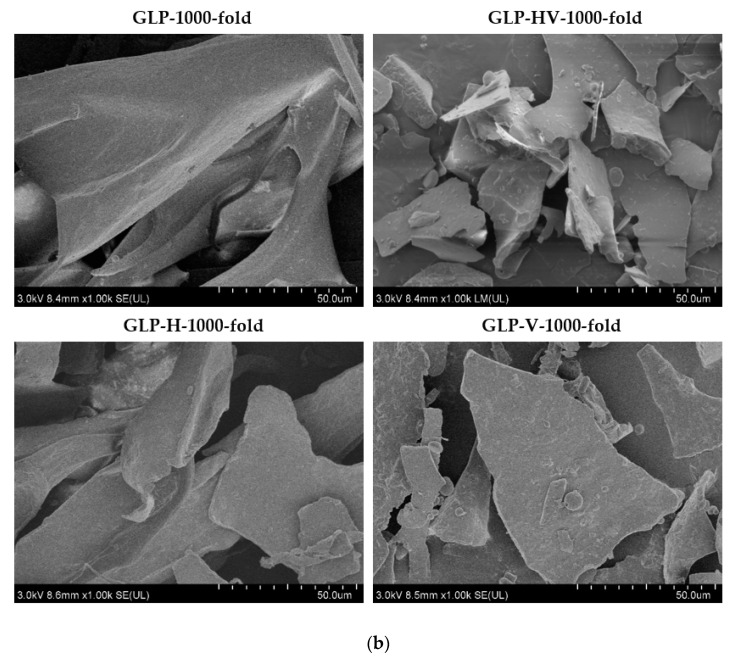
The surface morphology analysis of GLP and its degradation products. Note: (**a**) The surface morphology analysis of GLP, GLP-HV, GLP-H, GLP-V with the magnification of 200-fold; (**b**) The surface morphology analysis of GLP, GLP-HV, GLP-H, GLP-V with the magnification of 1000-fold.

The surface morphology analysis of GLP and its degradation products were explored by scanning electron microscope (SEM). The SEM images of GLP, GLP-HV, GLP-H, and GLP-V were observed in Figure 4. The SEM images at 200-fold (Figure 4a) and 1000-fold (Figure 4b) magnification indicated that the surface morphology of GLP was smooth, compact, and flaky. After the degradation with H_2_O_2_ and Vc, the polysaccharide structure was damaged, loose, and broken. The structure of GLP-V (treated by Vc) was damaged and became irregular (in the shapes of strips and flakes), but the surface was also smooth, compared with GLP. However, the structure of polysaccharide treated by H_2_O_2_ was broken, and its surface became rough and loose (GLP-H). The effect of H_2_O_2_-Vc degradation was more obvious, the structure was the most loose, and the damage for surface structure was the most serious, but the size of fragments was relatively uniform. The polysaccharide structure degraded by H_2_O_2_-Vc was more fragmented and looser. The morphologies of the polysaccharide produced by H_2_O_2_ degradation alone were similar to those treated by H_2_O_2_-Vc degradation, but the structures of the polysaccharide produced by H_2_O_2_-Vc were more uniform and fragmented. The probable reason was that H_2_O_2_ had a major effect on the structure of polysaccharide, while Vc played a supplementary role. Vc could promote H_2_O_2_ to generate more free radicals, breaking glyosidic bonds, so that the structure of polysaccharide became loose and broken [16]. 

### 2.9. The Inhibition Effect on α-glucosidase 

**Figure 5:**
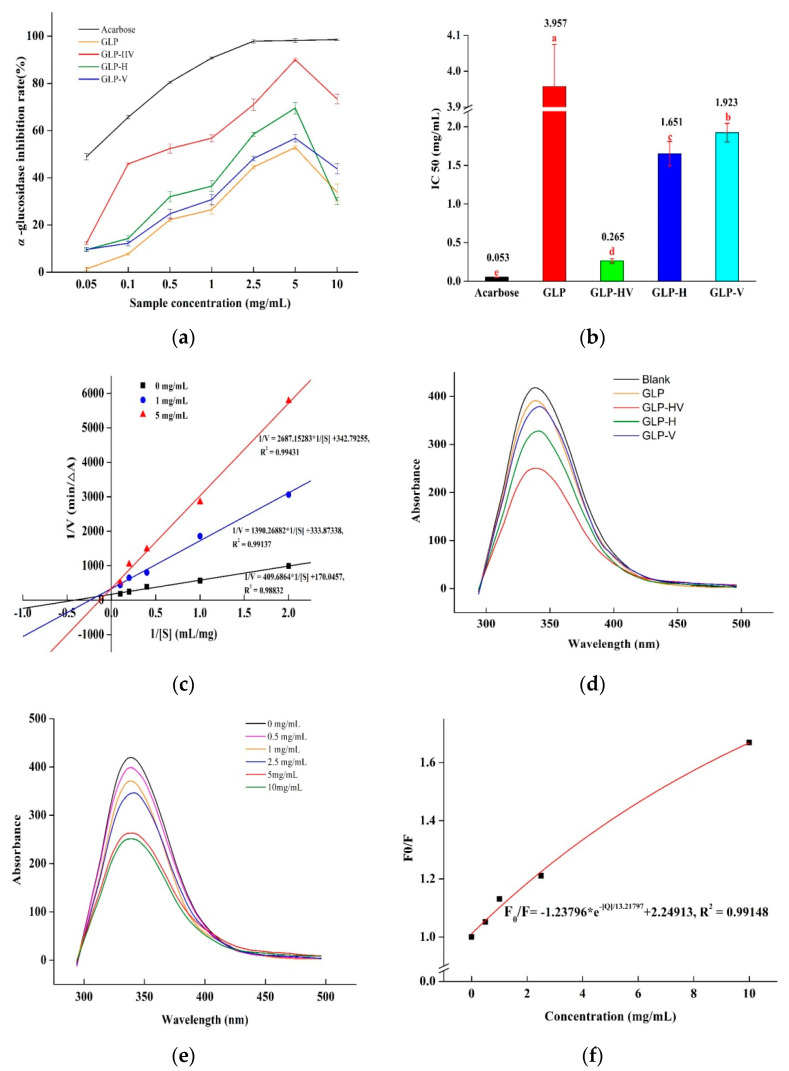
The inhibition effect of GLP and its degradation products on α-glucosidase. Note: (**a**) The α-glucosidase inhibition rate of GLP, GLP-HV, GLP-H, and GLP-V; (**b**) The IC_50_ value of α-glucosidase inhibition rate of GLP, GLP-HV, GLP-H, and GLP-V; (**c**) The Lineweaver–Burk curve of α-glucosidase inhibitory dynamics of GLP, GLP-HV, GLP-H, and GLP-V; (**d**) The fluorescence spectrum analysis on α-glucosidase of GLP, GLP-HV, GLP-H, and GLP-V; (**e**) The fluorescence spectrum analysis on α-glucosidase of GLP-HV; (**f**) The Stern-Volmer curve for fluorescence quenching of GLP-HV on α-glucosidase a, b, c, d and e represented significant differences among groups, and *p* < 0.05 indicates significant differences.

α-glucosidase is a key enzyme in hydrolysis of carbohydrate which can decompose carbohydrates into glucose. The inhibition of α-glucosidase activity could reduce the production of glucose, slow down the speed of glucose to enter the blood, and then decrease postprandial blood glucose [22]. In addition, this inhibition reduces the absorption of carbohydrates by the digestive tract and intestines, stimulates insulin-dependent glucose uptake, and reduces inflammation, thereby improving the symptoms of diabetes [31]. Acarbose, an α-glucosidase inhibitor, is a complex oligosaccharide with good inhibitory effect on α-glucosidase, hence it was selected as the positive control in this experiment [26]. Figure 5 displayed that GLP and its degradation products were able to inhibit α-glucosidase activity. In particular, GLP-HV exhibited an appreciable α-glucosidase inhibition, with inhibition rate of 89.98% as the content was 5 mg/mL (Figure 5a). At the same concentration, the inhibition rates of GLP, GLP-H, and GLP-V were 52.85%, 69.53%, and 56.76%, respectively, which significantly were inferior to GLP-HV. In the figure, it was clear that the inhibition rate was dose-dependent for all samples within a certain concentration range. Liao et al. [25] attained the α-glucosidase inhibition rate of GLP degradation product, treated by 9 mM H_2_O_2_-Vc for 2 h, was less than 70%, which was lower than GLP-HV (89.98%) at 5 mg/mL. It pointed out that H_2_O_2_-Vc concentration and the reaction time of degradation played a vital effect on the hypoglycemic activity of polysaccharide. Under different degradation conditions, the molecular weight, monosaccharide composition, and other structural characteristics of the obtained polysaccharide degradation products were obviously diverse, which resulted in the difference of hypoglycemic activity in vitro. In Figure 5b, the IC_50_ value of acarbose (control), GLP, GLP-HV, GLP-H, and GLP-V were 0.053, 3.957, 0.265, 1.651, and 1.923 mg/mL, which explained the inhibition effect on α-glucosidase of these samples from high to low was acarbose > GLP-HV > GLP-H > GLP-V > GLP. 

In Figure 5c, the Lineweaver–Burk curve of different concentrations of GLP-HV intersected in the second quadrant. From Table 6, when concentrations were 0, 1, 5 mg/mL, the *Km* values were 2.409, 4.164, and 7.839 mg/mL, and *Vmax* values were 0.005881, 0.002995, and 0.002917 mg/mL, respectively. *Km* was enhanced and *Vmax* was declined as the sample concentration increased, which were accorded with the mix of competitive and non-competitive inhibition, respectively. All of these results demonstrated that inhibition type of GLP-HV on α-glucosidase was the mix of competitive and non-competitive. The mechanism may be that samples would bind to the active sites of the enzyme, reducing the activity of the enzyme and preventing the combination of the enzyme to the substrate. On the other hand, the sample interacted with groups outside the active center of the enzyme, which had no direct inhibition effect on the enzyme, but can inhibit the release of products combined by enzyme and substrate, so as to achieve the inhibition effect. Cao et al. [32] studied the α-glucosidase inhibition effect of *Lentinus edodes* mycelia polysaccharide; the inhibition type also was mixed-type manner, which was in accordance with our results. Polysaccharides from different materials may have diverse types of α-glucosidase inhibition. Zhao et al. [33] found that the inhibition type was a competitive mode on α-glucosidase affected by polysaccharide from *Ribes nigrum* L. 

Figure 5d reflected the fluorescence intensity on α-glucosidase from GLP, GLP-HV, GLP-H, and GLP-V, which confirmed that all of the samples had fluorescence quenching effect to some extent. The fluorescence intensity became weakened, and the phenomenon of red shift occurred after the addition of samples. GLP-HV performed the best impact on the fluorescence intensity, and the second was GLP-H. GLP and GLP-V exhibited the similar effect, which were a little bit worse than the first two samples. Therefore, we chose GLP-HV with series of concentrations to observe the effect on fluorescence intensity of α-glucosidase (Figure 5e). The degree of redshift was bigger and bigger as the concentration increased (the concentration from 0 to 10 mg/mL), observed by Figure 5e and the value of *F* (Table 6). It demonstrated that the bigger content of GLP-HV displayed the stronger combination with the luminophore groups (tryptophan, tyrosine, and phenylalanine) of α-glucosidase. It was also possible that the combination of samples with the groups of the enzyme may cause changes of the environment around the luminescent groups, thus affecting the luminescence intensity. Calculating by fluorescence quenching Stern–Volmer equation, the fluorescence parameters on α-glucosidase of GLP-HV were showed in Table 7 and Figure 5f. The quenching constant *Ksv* and *Kq* were 0.07566 L/mol and 7.566 × 10^6^ L/mol/s, respectively. The value of *Ksv* was poorer than the other polysaccharide from literatures [34]. *Kq* was lower than 2.0 × 10^10^ L/mol/s [35], the maximum collision rate constant of quenchers caused by large biomolecules. On the other hand, the quenching curve between *F*_0_/*F* and *[Q]* almost showed the linear relationship. As a result, the fluorescence quenching type of GLP-HV to enzyme may be dynamic quenching, but the quenching effect was weak, according to the value of *Ksv*. *Ka* was the binding constant of GLP-HV with α-glucosidase, that could be calculated from the intercept of lg((*F*_0_ − *F*)/*F*) versus lg*[Q]* curve. Such data are summarized in Table 7: *Ka* was 9.5082 L/mol, and the number of binding sites (n) was 0.8137 (close to 1). We noticed that the value of *Ka* was relatively low, and the value of n was only 1, which indicated that the binding effect of the GLP-HV to the α-glucosidase was weak and there was only one binding site.

According to the results of inhibition rate, IC_50_, inhibition kinetics and fluorescence spectrum analysis, GLP-HV had a good inhibition effect on α-glucosidase in a dose-dependent manner, which was the mixed type of competitive and non-competitive. It had a certain quenching effect on fluorescence, which may be dynamic quenching. GLP-HV may have the potential to be developed as new hypoglycemic agents. 

## 3. Discussion

The degradation degree of polysaccharide was related to H_2_O_2_-Vc concentration, degradation time and temperature according to single factor and response surface experiments. H_2_O_2_-Vc concentration in the system was very small, and the degradation time was short, which had the advantages of rapid reaction, high efficiency, and low cost. The Mw of H_2_O_2_ and Vc were both lower than 300 g/mol, respectively, which can be removed by dialysis without introducing impurities and interfering with the activity of polysaccharide. This method is safe and reliable that can be used in industrial production. The total sugar and protein content were not changed after degradation, indicating that H_2_O_2_-Vc did not introduce other impurities. In addition, the reducing content of polysaccharide was higher after degradation, and its lower Mw, exhibiting the effective degradation of H_2_O_2_-Vc. And the degradation effect was better than fermentation, and similar to HPT treatment (high temperature and pressure combined with Vc) from the comparison of molecular weight [12,15].

Polysaccharide degradation is attributed to the breaking of glyosidic bonds, resulting in changes in monosaccharide composition. Various degradation methods act on different glyosidic bonds, so the breakage of glyosidic bonds’ location and number may be diverse, resulting in differences in the amount and content of monosaccharides exposed [11,20,21]. Xu et al. [13] found that the glucose and galactose were changed after degradation by HCl (the content of galactose was 35.9%) and enzyme (the content of galactose was 68.9%, respectively), compared with GLP (56.6%). It might be that the positions and quantities of glyosidic bonds destroyed by diverse degradation methods were different, leading to changes in monosaccharides content. However, the ratio of main monosaccharide components GLP-H (24.63% glucose and 66.39% galactose) and GLP-V (24.43% glucose and 69.64% galactose) were changed a little, compared with GLP, and exhibited the same variation. Moreover, the level of ribose and xylose enhanced, but rhamnose content declined after degradation. This phenomenon may be due to the position of glyosidic bond affected by different degradation ways. Alone, H_2_O_2_ or Vc treatment may break the similar position or numbers of glyosidic bond. They may cut the glyosidic bonds on both sides of galactose, promoting the produce of galactose, compared with H_2_O_2_-Vc treatment. The degradation of polysaccharide led to the change of monosaccharide composition, which may be the main reason for the change of glucosidase inhibition effect by polysaccharide and its production products. 

The SEM results were consistent with the change of reducing sugar and molecular weight, which exhibited that H_2_O_2_-Vc obviously degraded the *Gracilaria lemaneiformis* polysaccharide effectively, thus could break its structure, and changed its surface morphology. Furthermore, H_2_O_2_-Vc treatment exhibited the better degradation effect than alone H_2_O_2_ or Vc treatment, might be due to the differences in the distance and degree of crosslinking between molecules. Gong et al. [11] also found that GLP presented the thick slices image with flat and smooth surface, and then changed into thin, lacerating, and rough after degradation by UV-H_2_O_2_. However, the polysaccharide structure was more fragmented and looser degraded by H_2_O_2_-Vc. Perhaps Vc reacted with H_2_O_2_ to produce more free radicals, which had a stronger effect on the polysaccharide molecules. The Mw of polysaccharides dreaded by H_2_O_2_ or Vc were only slightly less than GLP, and H_2_O_2_-Vc exhibited the excellent degradation effect with the minimum Mw. This finding validated the SEM result.

α-glucosidase is one of the hydrolases in the digestive tract, which is closely related to hypoglycemic activity [32]. The inhibition effect of polysaccharide on α-glucosidase can reduce the production of glucose in the blood and thus change the carbohydrate metabolism. Therefore, the inhibitory effect of polysaccharide on α-glucosidase can reflect the hypoglycemic effect in vitro. From the results of the inhibition effect of α-glucosidase, the polysaccharide treated by H_2_O_2_-Vc enhanced the hypoglycemic activity in vitro, compared with H_2_O_2_ or Vc treatment alone. GLP-HV presented higher galactose, ribose, and xylose than GLP, and lower glucose and rhamnose. Both GLP-H and GLP-V expressed similar changes with GLP-HV, but the effect were not as significant as GLP-HV. The degraded polysaccharides showed better inhibition, especially GLP-HV. It possibly was related to these changes in monosaccharide compositions. Cao et al. [32] exerted the outstanding inhibition on α-glucosidase by *Lentinus edodes* mycelia polysaccharide containing mannose, arabinose, galactose, xylose, and rhamnose. Combined with the results of molecular weight, GLP-HV had the lower Mw than GLP, GLP-H, and GLP-V. For another, the Mw of GLP-V was smaller than GLP and GLP-H, but its inhibition rate on α-glucosidase was worse than GLP-H and higher than GLP. Therefore, the good inhibition effect of GLP-HV may not only be due to the low molecular weight and monosaccharide composition, but also to the connection mode of glycoside chains, which needs to be verified by subsequent studies. Lv et al. [30] researched the backbone of WXA-1 (polysaccharide from wheat bran) was →4)-β-D-Xylp-(1→, which was substituted at O-3 positions by arabinose, glucose, and galactose residues, while the backbone of AXA-1 (polysaccharide from wheat bran) was → 4)-β-D-Xylp-(1→, which was mainly substituted at O-3 positions by arabinose. AXA-1 exhibited a stronger inhibitory effect on the activities of α-amylase and α-glucosidase compared with WXA-1. When the concentration range of GLP-HV was 0–5 mg/mL, the inhibition rate of α-glucosidase was dose-dependent, and the higher concentration played the higher inhibition rate. When the concentration was from 5 to 10 mg/mL, the inhibition rate decreased. However, fluorescence quenching effect of GLP-HV with 10 mg/mL was a little bit stronger than 5 mg/mL, which was contrary to the results of inhibition rate. The probable reason may that the inhibition type was the mix of competitive and non-competitive. GLP-HV inhibited the products of enzyme-substrate interaction rather than directly binding to the enzyme active groups. Furthermore, the strength of fluorescence quenching cannot fully represent the inhibitory effect on the enzyme. The fluorescence spectrum of α-glucosidase under this condition was due to the existence of luminescent groups such as tryptophan, tyrosine, and phenylalanine. Changes of the groups and their environment led to the changes in fluorescence intensity. The weak binding ability between GLP-HV and the luminescent group or the weak influence on the environment of the luminescent group cannot fully explain the weak inhibitory activity of the sample.

The method of hot water extraction and alcohol precipitation is a suitable extraction method for polysaccharide, with high total sugar content and without protein. H_2_O_2_-Vc is also an excellent degradation method, which has the advantages of high efficiency, low cost, and no by-products. Low molecular weight polysaccharide with high hypoglycemic activity could be obtained by H_2_O_2_-Vc degradation. Polysaccharide degradation products showed the inhibition on α-glucosidase, and GLP-HV presented the best effect in a dose-dependent manner, which was the mixed type of competitive and non-competitive. It had a certain quenching effect on fluorescence of α-glucosidase, which may be dynamic quenching. The best inhibition effect on α-glucosidase may be related to its molecular weight, monosaccharide composition and other factors.

## 4. Materials and Methods

### 4.1. Materials and Chemicals

*Gracilaria lemaneiformis* was purchased from Nan’ao Island (Shantou, Gguangdong, China), the Coomassie Bright Blue kit was purchased from Shanghai Biyuntian Biotechnology Co., LTD (Shanghai, China); vitamin C (Vc) and α-glucosidase were purchased from Shanghai Yuanye Biotechnology Co., LTD (Shanghai, China); P-nitrobenzene-α-d-glucoside (PNPG) was purchased from Shanghai Maclin Biochemical Technology Co., LTD (Shanghai, China).

### 4.2. Preparation of GLP

*Gracilaria lemaneiformis* was cleaned many times to remove impurities, dried in an oven at 50 °C, crushed and sifted through 40 mesh to obtain uniform powder. The powder was soaked on a shaking table (50 °C) with an ethanol volume of nine times to remove pigment, fat, and alcohol-soluble impurities. After 24 h, the filter residue was extracted and placed in a drying oven at 50 °C for drying. The GLP was extracted by hot water and precipitated by ethanol according to the previous reported methods with some modifications [11]. After 30 min of ultrasonic wall breaking, *Gracilaria lemaneiformis* was extracted with water at a ratio of 1:45 (*w*/*v*) at 90 °C for 4 h under oscillation, and then cooled to room temperature to add 1% papain and 0.5% cellulase (*w*/*v*). The mixture was incubated at 60 °C for 2 h under oscillation, and then rapidly heated in boiling water for 10 min to denature the papain and cellulase. After cooling to room temperature, the mixture was centrifuged at 8000 rpm for 15 min, and the supernatant was collected and condensed to one third of the original volume by rotary evaporation (60 °C). The concentrated solution was precipitated at 4 °C for 12 h, centrifuged for precipitation, filtered with 200 mesh gauze, and then dialyzed (7 kDa) at 4 °C for 72 h, and pure water was changed once at 4 h. After freeze-drying, GLP values were obtained. 

### 4.3. Degradation of GLP with H_2_O_2_-Vc

#### 4.3.1. Single-Factor Experiment

The degradation was adopted based on the reported method [10]. GLP was degraded by H_2_O_2_-Vc (mole ratio of H_2_O_2_ and Vc was 1:1). In brief, GLP (5 mg/mL) and H_2_O_2_-Vc at different concentrations (5, 10, 15, 20, and 25 mM) were added directly into the solution. Degradation times were 0.1, 0.3, 0.5, 0.7, and 0.9 h, and the temperature were maintained at 30, 40, 50, 60, and 70 °C. Reducing sugar content and α-glucosidase inhibition rate were used as screening indexes.

#### 4.3.2. Response Surface Analysis

According to the single-factor experimental results, the response surface method was designed and implemented using the Box–Behnken Design (BBD) principle of Design-Expert 8.0.6 software, and the response surface results were fitted. 

#### 4.3.3. Preparation of Degradation Products from GLP

The polysaccharide was degraded by the optimal process obtained by the results of single-factor experiment and response surface analysis. The degradation product was neutralized (pH 7.0) with 1 M NaOH, concentrated to 1/3 volume at 60 °C using a rotary evaporator, and precipitated at 4 °C for 12 h with absolute ethanol (1:4, *v*/*v*). After centrifuging (8000 rpm, 15 min), the precipitation was dissolved with pure water in the magnetic blender, and then dialyzed (300 Da) at 4 °C for 48 h, and pure water was changed once at 4 h. After freeze-drying, the degradation product was obtained, named GLP-HV (*Gracilaria lemaneiformis* polysaccharide degraded by H_2_O_2_-Vc). Only H_2_O_2_ in the same concentration was used for polysaccharide, the other conditions were the same, the sample was obtained, named GLP-H (*Gracilaria lemaneiformis* polysaccharide degraded by H_2_O_2_). Only Vc in the same concentration was used for polysaccharide, the other conditions were the same, the sample was obtained, named GLP-V (*Gracilaria lemaneiformis* polysaccharide degraded by Vc). GLP-H and GLP-V were the controls of GLO-HV.

### 4.4. Analysis of Chemical Characterizatics

#### 4.4.1. Determination of Total Sugar, Reducing Sugar and Protein Content

Total sugar, reducing sugar, and protein content were determined by phenol-sulfuric acid method with D-glucose as the standard compound, DNS (3,5-Dinitrosalicylic acid) method with D-glucose as the standard compound, and Coomassie Bright Blue kit.

#### 4.4.2. Determination of Monosaccharide Composition

Monosaccharide composition was determined using high performance liquid chromatography (LC-20AD) according to the method reported by Kang et al. [9], with a slight modification. The analysis conducted with a drift tube temperature of 30 °C, wavelength of 250 nm, using Xtimate C18 column (4.6 mm × 250 mm, 5 µm) at 30 °C for 50 min. The monosaccharides were eluted using 0.05 M potassium dihydrogen phosphate (PH 6.7)-acetonitrile mobile phase (83:17) at a flow rate of 1 mL/min.

Derivatives of standard products: After the monosaccharide standards were dissolved in water, 250 uL 0.6 mol/L NaOH and 500 uL 0.4 mol/L PMP-methanol were added and reacted at 70 °C for 1 h. Subsequently, the solution was cooled in cold water for 10 min, 500 uL 0.3 mol/L HCl was added for neutralization, and 1 mL chloroform was added and mixed. Centrifugation was performed at 3000 r/min for 10 min. The supernatant was carefully taken and extracted three times. 

Hydrolysis and derivatization of samples: appropriate amounts of samples were accurately weighed, 2 mL 2 mol/L trifluoroacetylacetone (TFA) was added and acidolized at 120 °C for 4 h. TFA was blow-dried with nitrogen and redissolved with 2 mL water. The derivatization procedure of the hydrolyzed sample solution was consistent with that of the standard. 

#### 4.4.3. Determination of Molecular Weight 

Molecular weight was determined by Gel Permeation Chromatography (Shimadzu, Japan), referred to the report [36], with a slight modification. The analysis carried out on differential refractive index detector of RID-20A with a drift tube temperature of 35 °C, using TSKgel GMPWXL column for 25 min. The molecular weight was eluted using 0.1 M sodium nitrate (NaNO_3_) and 0.06% Sodium azide (NaN_3_) at a flow rate of 0.6 mL/min.

#### 4.4.4. UV-Visible Spectroscopy 

The method was referred to the previous research [37]. The absorption spectra of sample solutions (0.5 mg/mL) were measured using a UV-vis spectrophotometer (UV2550, Shimadzu, Japan), with the wavelength ranging from 200 to 700 nm with an interval of 1 nm.

#### 4.4.5. I_2_-KI Test

A polysaccharide and degradation products solution (2.0 mg/mL, 2.0 mL) was mixed with I_2_-KI reagent (0.8 mL, containing 0.2% KI and 0.02% I_2_, *w*/*v*) for 10 min. The absorbance was detected by a UV-vis spectrophotometer (UV2550, Japan) with the range of 300–700 nm [38].

#### 4.4.6. Congo Red Test 

Polysaccharide and degradation products solution (2 mg/L, 2.0 mL) were prepared, and 2 mL of 80 uM Congo red was added, and then 1 M NaOH was dropped to make the concentration of NaOH in different solutions vary from 0 to 0.5 mol/L. UV-vis spectrophotometer scanning (400–700 nm) was conducted to determine the maximum absorption wavelength under various concentration gradients of NaOH [33].

#### 4.4.7. Scanning Electron Microscope Analysis (SEM)

The morphology of GLP, GLP-HV, GLP-H, and GLP-V were analyzed by SEM (SU 8010, Hitachi, Japan) [38]. These samples were dipped in a small amount of powder with conductive tape and pasted on the sample table. After spraying gold for 30–60 s, the samples were vacuumized and tested on the machine. The images were taken by SU 8010 Hitachi scanning electron microscope and the magnifications were 1000-fold and 200-flod, respectively.

### 4.5. The Inhibition Effect on α-Glucosidase

The inhibition effect on α-glucosidase was detected according to the previous report [26], with some modifications.

The inhibition rate: 30 μL polysaccharide and degradation products solution (0.1, 0.5, 1, 2.5, 5, 10 mg/mL), mixed with 100 μL phosphate buffer solution (PBS, 0.1 mol/L, pH 6.8,) and 30 μL α-glucoside enzyme solution (0.5 U/mL, pH 6.8, prepared by PBS) in 96-well plates, which incubated at 37 °C for 15 min. 30 μL PNPG solution (10 mmol/L, prepared by PBS) was mixed into the reaction mixture and incubated at 37 °C for 20 min. Then measured the absorbance value at 405 nm, and calculated the inhibitory activity according to the following formula:

Inhibition rate/% = ((A_3_ − A4) − (A_1_ − A_2_))/(A_3_ − A_4_) × 100, (1)

A_1_: Samples and enzyme; A_2_: The enzyme was replaced by PBS; A_3_: Samples are replaced with pure water; A_4_: Samples and enzyme were replaced with pure water and PBS, respectively.

Inhibition kinetics: The sample concentration was 0, 1, and 5 mg/mL, and the concentration of PNPG was 1, 2.5, 5, 7.5, and 10 mM, respectively. The reaction time was 20 min, and the determination was performed every 2 min. The Lineweaver–Burk curve was drawn by double reciprocal plotting method with reciprocal of substrate concentration as abscissa and reciprocal of reaction rate as ordinate, which calculated the Michaelis constant (*Km*), the maximum reaction rate (*Vmax*), according to the following formula:

1/*v* = *Km*/*Vmax* × 1/(*[S]*) + 1/*Vmax*, (2)

*V*: initial reaction rate; *Vmax*: the maximum reaction rate; *[S]*: the concentration of PNPG; *Km*: the Michaelis constant.

Fluorescence spectrum analysis: The fluorescence spectrum analysis was studied according to literature [31] with some modifications. The excitation wavelength was 280 nm, the emission wavelength was 290–500 nm, and the slit width was 5 nm. 0.5 mL samples solution (5 mg/mL) and 2.5 mL α-glucosidase (5 U/mL) were taken, and the fluorescence spectrum was measured under these conditions after shaking and mixing, and the samples were replaced by PBS as blank. The samples with the best activity were prepared with concentrations of 0.1, 0.5, 1, 2.5, 5 and 10 mg/mL, and the fluorescence spectra were determined under the same conditions. The equation of Stern–Volmer was used to express the fluorescence quenching:

*F*_0_/*F* = 1 + *Ksv [Q]* = 1 + *Kq* τ_0_
*[Q]*, (3)

*F*_0_/*F* = e^(*Ksv[Q]*), (4)

*Ksv* = *Kq*τ_0_, (5)

lg ((*F*_0_ − *F*)/*F*) = lg*Ka* + nlg*[Q]*, (6)

*F*_0_: fluorescence intensity of α-glucosidase without samples; *F*: fluorescence intensity of α-glucosidase with samples; *[Q]*: the concentration of samples; *τ_0_*: the average life of fluorescent substances without quenching agent is generally 10^−8^; *Kq*: quenching rate constant; *Ksv*: quenching constant of Stern–Volmer. *Ka*: the binding constant between samples and α-glucosidase; n: the binding-site number.

### 4.6. Statistical Analysis

Data are expressed as means ± standard (SD). Duncan’s multiple range test was applied to identify differences between the mean values for each group by IBM SPSS software (version 22). *p* < 0.05 was considered to represent statistical significance. The degradation experimental design and analysis was performed using Design-Expert 8.0.6. IC_50_ was calculated by IBM SPSS software 22. Figures were finished by Origin-Pro 8.5.

## 5. Conclusions

The optimal conditions for H_2_O_2_-Vc degradation were as follows: H_2_O_2_-Vc concentration was 18.7 mM, reaction time was 0.5 h, and reaction temperature was 56 °C. The total sugar content of GLP, GLP-HV, GLP-H and GLP-V were 98.77%, 98.43%, 97.28%, and 97.5%, respectively, and their reducing sugar were 2.47%, 46.92%, 1.95%, and 50.2%, respectively. Moreover, the Mw was reduced after degradation, and the monosaccharides were mainly glucose and galactose before and after degradation. In addition, GLP and its degradation products did not have protein, starch, and triple helix structure. The degradation method of H_2_O_2_-Vc is feasible, and no by-products can be produced. The SEM analysis demonstrated that H_2_O_2_-Vc made the structure loose and broken. All samples showed the inhibition on α-glucosidase, and GLP-HV presented the best effect in a dose-dependent manner, which was the mixed type of competitive and non-competitive. It had a certain quenching effect on fluorescence of α-glucosidase, which may be dynamic quenching. The polysaccharide degraded by H_2_O_2_-Vc, with low Mw, exerted the good inhibition effect on glucosidase activity.

## Figures and Tables

**Table 1:** Factor level coding.

Factor	Level		
	−1	0	1
A H_2_O_2_-Vc concentration/mM	15	20	25
B Time/h	0.3	0.5	0.7
C Temperature/°C	40	50	60

**Table 2:** Experimental design and results for response surface analysis.

Code	A:H_2_O_2_-Vc Concentration/mM	B: Time/h	C:Temperature/°C	Y:Reducing Sugar Content/%
1	0	0	0	41.6564
2	0	0	0	40.6566
3	1	0	−1	32.9644
4	1	0	1	35.5119
5	0	1	1	39.2347
6	0	0	0	40.6399
7	−1	0	1	40.1668
8	0	0	0	41.0565
9	0	−1	−1	34.0254
10	0	−1	1	38.7351
11	−1	1	0	36.8824
12	1	1	0	36.1679
13	−1	0	−1	34.2537
14	1	−1	0	32.7301
15	0	0	0	40.0567
16	−1	−1	0	36.8499
17	0	1	−1	36.5345

**Table 3:** ANOVA of regression equation.

Source	Sun of Squares	df	Mean Square	*F* Value	*p* Value	Significance
Model	139.59	9	15.51	63.28	<0.0001	**
A-H_2_O_2_-Vc concentration	14.52	1	14.52	59.25	0.0001	**
B-Time	5.25	1	5.25	21.41	0.0024	**
C-Temperature	31.48	1	31.48	128.46	<0.0001	**
AB	2.90	1	2.90	11.83	0.0108	*
AC	2.83	1	2.83	11.55	0.0115	*
BC	1.01	1	1.01	4.12	0.0820	
A^2^	45.35	1	45.35	185.04	<0.0001	**
B^2^	14.78	1	14.78	60.31	0.0001	**
C^2^	13.75	1	13.75	56.10	0.0001	**
Residual	1.72	7	0.25			
Lack of Fit	0.32	3	0.11	0.30	0.8223	
Pure Error	1.40	4	0.35			
Cor Total	141.31	16				

Note: * significant difference (*p* < 0.05); ** extremely significant difference (*p* < 0.01).

**Table 4:** The content of total sugar, reducing sugar and protein from GLP and its degradation products.

Indexes		GLP	GLP-HV	GLP-H	GLP-V
Total sugar	Content (%)	98.77 ± 5.94 a	98.43 ± 3.21 a	97.28 ± 2.63 a	97.5 ± 1.56 a
	Standard curve	y = 2.4623x + 0.0516, R^2^ = 0.9939			
Reducing sugar	Content (%)	2.47 ± 0.03 c	46.92 ± 2.38 b	1.95 ± 0.15 c	50.2 ± 1.00 a
	Standard curve	y = 0.8185x − 0.0258, R^2^ = 0.9999			
Protein	Content (%)	ND	ND	ND	ND
	Standard curve	y = 0.6288x + 0.5582, R^2^ = 0.9902			

Note: a, b, and c represent significant differences among groups, and *p* < 0.05 indicates significant differences.

**Table 5:** The Molecular weight from GLP and its degradation products.

Molecular Weight (Da)	GLP	GLP-HV	GLP-H	GLP-V
Number average molecular weight(Mn) (Da)	10,250	6695	17,508	21,224
Weight average molecular weight(Mw) (Da)	1,478,524	16,245	1,329,838	1,000,630
Mw/Mn	144.24	2.43	75.96	47.15

**Table 6:** The inhibitory kinetic constant on α-glucosidase of GLP-HV.

*[S]* (mg/mL)	*V max* (mg/mL·min^−1^)	*Km* (mg/mL)	Inhibition Type
0	0.0059	2.409	The mix of competitive and non-competitive
1	0.0030	4.164	
5	0.0029	7.839	

**Table 7:** The fluorescence parameter on α-glucosidase of GLP-HV.

*[Q]* (mg/mL)	*F*	*F*_0_/*F*	*Ksv* (L/mol)	*Kq* (L/mol/s)	*Ka* (L/mol)	*n*
0	419	1	0.07566	7.566 × 106	9.5082	0.8137
0.5	399	1.0518				
1	371	1.1309				
2.5	346	1.2107				
5	263	1.5930				
10	251	1.6690				
equation of curve	*F*_0_/*F*= −1.23796∗e^−[Q]/13.21797^ + 2.24913, R^2^ = 0.99148				lg((*F*_0_ − *F*)/*F*) = 0.8137∗lg*[Q]*−0.9781, R^2^ = 0.9788	

## Data Availability

Data is contained within the article.

## References

[1] Yu Y.-Y., Chen W.-D., Liu Y.-J., Niu J., Chen M., Tian L.-X. (2016). Effect of different dietary levels of Gracilaria lemaneiformis dry power on growth performance, hematological parameters and intestinal structure of juvenile Pacific white shrimp (*Litopenaeus vannamei*). Aquaculture.

[2] Long X., Hu X., Liu S., Pan C., Chen S., Li L., Qi B., Yang X. (2021). Insights on preparation, structure and activities of Gracilaria lemaneiformis polysaccharide. Food Chem. X.

[3] Fisheries Administration of Ministry of Agriculture and Rural Affairs (2020). China Fishery Statistical Yearbook.

[4] Li X., Huang S., Chen X., Xu Q., Ma Y., You L., Kulikouskaya V., Xiao J., Piao J. (2020). Structural characteristic of a sulfated polysaccharide from Gracilaria Lemaneiformis and its lipid metabolism regulation effect. Food Funct..

[5] Sun X., Duan M., Liu Y., Luo T., Ma N., Song S., Ai C. (2018). The beneficial effects of Gracilaria lemaneiformis polysaccharides on obesity and the gut microbiota in high fat diet-fed mice. J. Funct. Foods.

[6] Fan Y., Wang W., Song W., Chen H., Teng A., Liu A. (2012). Partial characterization and anti-tumor activity of an acidic polysaccharide from Gracilaria lemaneiformis. Carbohydr. Polym..

[7] Chen M.-Z., Xie H.-G., Yang L.-W., Liao Z.-H., Yu J. (2010). In vitro anti-influenza virus activities of sulfated polysaccharide fractions from Gracilaria lemaneiformis. Virol. Sin..

[8] Wang X., Zhang Z., Zhou H., Sun X., Chen X., Xu N. (2019). The anti-aging effects of Gracilaria lemaneiformis polysaccharide in Caenorhabditis elegans. Int. J. Biol. Macromol..

[9] Kang Y., Wang Z.-J., Xie D., Sun X., Yang W., Zhao X., Xu N. (2017). Characterization and Potential Antitumor Activity of Polysaccharide from Gracilariopsis lemaneiformis. Mar. Drugs.

[10] Chen S., Liu H., Yang X., Li L., Qi B., Hu X., Ma H., Li C., Pan C. (2020). Degradation of sulphated polysaccharides from Grateloupia livida and antioxidant activity of the degraded components. Int. J. Biol. Macromol..

[11] Gong Y., Ma Y., Cheung P.C.-K., You L., Liao L., Pedisić S., Kulikouskaya V. (2021). Structural characteristics and anti-inflammatory activity of UV/H2O2-treated algal sulfated polysaccharide from Gracilaria lemaneiformis. Food Chem. Toxicol..

[12] Zhang X., Aweya J.J., Huang Z.-X., Kang Z.-Y., Bai Z.-H., Li K.-H., He X.-T., Liu Y., Chen X.-Q., Cheong K.-L. (2020). In vitro fermentation of Gracilaria lemaneiformis sulfated polysaccharides and its agaro-oligosaccharides by human fecal inocula and its impact on microbiota. Carbohydr. Polym..

[13] Xu X.-Q., Su B.-M., Xie J.-S., Li R.-K., Yang J., Lin J., Ye X.-Y. (2018). Preparation of bioactive neoagaroligosaccharides through hydrolysis of Gracilaria lemaneiformis agar: A comparative study. Food Chem..

[14] Jin M., Liu H., Hou Y., Chan Z., Di W., Li L., Zeng R. (2017). Preparation, characterization and alcoholic liver injury protective effects of algal oligosaccharides from Gracilaria lemaneiformis. Food Res. Int..

[15] Liu Q., Zhang Y., Shu Z., Liu M., Zeng R., Wang Y., Liu H., Cao M., Su W., Liu G. (2020). Sulfated oligosaccharide of Gracilaria lemaneiformis protect against food allergic response in mice by up-regulating immunosuppression. Carbohydr. Polym..

[16] Yan S., Pan C., Yang X., Chen S., Qi B., Huang H. (2021). Degradation of Codium cylindricum polysaccharides by H2O2-Vc-ultrasonic and H2O2-Fe2+-ultrasonic treatment: Structural characterization and antioxidant activity. Int. J. Biol. Macromol..

[17] Shen X., Liu Z., Li J., Wu D., Zhu M., Yan L., Mao G., Ye X., Linhardt R.J., Chen S. (2019). Development of low molecular weight heparin by H2O2/ascorbic acid with ultrasonic power and its anti-metastasis property. Int. J. Biol. Macromol..

[18] Shokri Z., Seidi F., Karami S., Li C., Saeb M.R., Xiao H. (2021). Laccase Immobilization onto Natural Polysaccharides for Biosensing and Biodegradation. Carbohydr. Polym..

[19] Chen H., Xiao Q., Weng H., Zhang Y., Yang Q., Xiao A. (2020). Extraction of sulfated agar from Gracilaria lemaneiformis using hydrogen peroxide-assisted enzymatic method. Carbohydr. Polym..

[20] Chen X., Li X., Sun-Waterhouse D., Zhu B., You L., Hileuskaya K. (2021). Polysaccharides from Sargassum fusiforme after UV/H2O2 degradation effectively ameliorate dextran sulfate sodium-induced colitis. Food Funct..

[21] Li J., Li S., Liu S., Wei C., Yan L., Ding T., Linhardt R.J., Liu D., Ye X., Chen S. (2019). Pectic oligosaccharides hydrolyzed from citrus canning processing water by Fenton reaction and their antiproliferation potentials. Int. J. Biol. Macromol..

[22] Nasab S.B., Homaei A., Pletschke B.I., Salinas-Salazar C., Castillo-Zacarias C., Parra-Saldívar R. (2020). Marine resources effective in controlling and treating diabetes and its associated complications. Process. Biochem..

[23] Lim J., Ferruzzi M.G., Hamaker B.R. (2022). Structural requirements of flavonoids for the selective inhibition of α-amylase versus α-glucosidase. Food Chem..

[24] Wen L., Zhang Y., Sun-Waterhouse D., You L., Fu X. (2017). Advantages of the polysaccharides from Gracilaria lemaneiformis over metformin in antidiabetic effects on streptozotocin-induced diabetic mice. RSC Adv..

[25] Liao X., Yang L., Chen M., Yu J., Zhang S., Ju Y. (2015). The hypoglycemic effect of a polysaccharide (GLP) from Gracilaria lemaneiformis and its degradation products in diabetic mice. Food Funct..

[26] Zheng Q., Jia R.-B., Ou Z.-R., Li Z.-R., Zhao M., Luo D., Lin L. (2021). Comparative study on the structural characterization and α-glucosidase inhibitory activity of polysaccharide fractions extracted from Sargassum fusiforme at different pH conditions. Int. J. Biol. Macromol..

[27] Wu S., Lu M., Wang S. (2017). Amylase-assisted extraction and antioxidant activity of polysaccharides from Gracilaria lemaneiformis. 3 Biotech.

[28] Liu Q.-M., Yang Y., Maleki S.J., Alcocer M., Xu S.-S., Shi C.-L., Cao M.-J., Liu G.-M. (2016). Anti-Food Allergic Activity of Sulfated Polysaccharide from Gracilaria lemaneiformis is Dependent on Immunosuppression and Inhibition of p38 MAPK. J. Agric. Food Chem..

[29] Ren Y., Zheng G., You L., Wen L., Li C., Fu X., Zhou L. (2017). Structural characterization and macrophage immunomodulatory activity of a polysaccharide isolated from Gracilaria lemaneiformis. J. Funct. Foods.

[30] Lv Q.-Q., Cao J.-J., Liu R., Chen H.-Q. (2021). Structural characterization, α-amylase and α-glucosidase inhibitory activities of polysaccharides from wheat bran. Food Chem..

[31] Fu M., Shen W., Gao W., Namujia L., Yang X., Cao J., Sun L. (2021). Essential moieties of myricetins, quercetins and catechins for binding and inhibitory activity against α-Glucosidase. Bioorganic Chem..

[32] Cao X., Xia Y., Liu D., He Y., Mu T., Huo Y., Liu J. (2020). Inhibitory effects of Lentinus edodes mycelia polysaccharide on α-glucosidase, glycation activity and high glucose-induced cell damage. Carbohydr. Polym..

[33] Zhao M., Bai J., Bu X., Yin Y., Wang L., Yang Y., Xu Y. (2021). Characterization of selenized polysaccharides from Ribes nigrum L. and its inhibitory effects on α-amylase and α-glucosidase. Carbohydr. Polym..

[34] Wang S., Li Y., Huang D., Chen S., Xia Y., Zhu S. (2022). The inhibitory mechanism of chlorogenic acid and its acylated derivatives on α-amylase and α-glucosidase. Food Chem..

[35] Bharathi D., Siddlingeshwar B., Krishna R.H., Kirilova E.M., Divakar D.D., Alkheraif A.A. (2021). Interaction of CuO and ZnO nanoparticles with 3-N-(N′-methylacetamidino) benzanthrone: A temperature dependent fluorescence quenching study. Inorg. Chem. Commun..

[36] Zhang S., Hong H., Zhang H., Chen Z. (2021). Investigation of anti-aging mechanism of multi-dimensional nanomaterials modified asphalt by FTIR, NMR and GPC. Constr. Build. Mater..

[37] Pattanayak S., Chakraborty S., Biswas S., Chattopadhyay D., Chakraborty M. (2018). Degradation of Methyl Parathion, a common pesticide and fluorescence quenching of Rhodamine B, a carcinogen using β-d glucan stabilized gold nanoparticles. J. Saudi Chem. Soc..

[38] Wang L., Li L., Gao J., Huang J., Yang Y., Xu Y., Liu S., Yu W. (2021). Characterization, antioxidant and immunomodulatory effects of selenized polysaccharides from dandelion roots. Carbohydr. Polym..

